# Heat Shock Protein 90 as Therapeutic Target for CVDs and Heart Ageing

**DOI:** 10.3390/ijms23020649

**Published:** 2022-01-07

**Authors:** Siarhei A. Dabravolski, Vasily N. Sukhorukov, Vladislav A. Kalmykov, Nikolay A. Orekhov, Andrey V. Grechko, Alexander N. Orekhov

**Affiliations:** 1 Department of Clinical Diagnostics, Vitebsk State Academy of Veterinary Medicine [UO VGAVM], 7/11 Dovatora Str., 210026 Vitebsk, Belarus; 2 Laboratory of Cellular and Molecular Pathology of Cardiovascular System, AP Avtsyn Research Institute of Human Morphology, 3 Tsyurupy Str., 117418 Moscow, Russia; vasily.sukhorukov@morfolhum.ru (V.N.S.); xxor2011@gmail.com (V.A.K.); 3 Laboratory of Medical Genetics, Russian Medical Research Center of Cardiology, Institute of Experimental Cardiology, 15-a 3-rd Cherepkovskaya Str., 121552 Moscow, Russia; 4 Laboratory of Angiopathology, Institute of General Pathology and Pathophysiology, Russian Academy of Medical Sciences, 125315 Moscow, Russia; 5 Institute for Atherosclerosis Research, 4-1-207 Osennyaya Str., 121609 Moscow, Russia; fuper@gmail.com (N.A.O.); a.h.opexob@gmail.com (A.N.O.); 6 Federal Research and Clinical Center of Intensive Care Medicine and Rehabilitology, 14-3 Solyanka Str., 109240 Moscow, Russia; avg-2007@yandex.ru

**Keywords:** heat shock protein, chaperone, cardiovascular diseases, atherosclerosis, ageing

## Abstract

Cardiovascular diseases (CVDs) are the leading cause of death globally, representing approximately 32% of all deaths worldwide. Molecular chaperones are involved in heart protection against stresses and age-mediated accumulation of toxic misfolded proteins by regulation of the protein synthesis/degradation balance and refolding of misfolded proteins, thus supporting the high metabolic demand of the heart cells. Heat shock protein 90 (HSP90) is one of the main cardioprotective chaperones, represented by cytosolic *HSP90a* and *HSP90b*, mitochondrial *TRAP1* and ER-localised *Grp94* isoforms. Currently, the main way to study the functional role of HSPs is the application of HSP inhibitors, which could have a different way of action. In this review, we discussed the recently investigated role of HSP90 proteins in cardioprotection, atherosclerosis, CVDs development and the involvements of HSP90 clients in the activation of different molecular pathways and signalling mechanisms, related to heart ageing.

## 1. Introduction

Cardiovascular diseases (CVDs) are the leading cause of death worldwide. CVDs are defined as a group of heart and blood vessels disorders, including coronary heart disease, peripheral arterial disease, pulmonary embolism, deep vein thrombosis, cerebrovascular diseases and others. In 2019, 32% of all global deaths were from CVDs, with stroke and heart attack causing 85% of these deaths [1]. Recently, mitochondria have emerged as a key player in the progression of CVDs, thus making mitochondria-targeted therapies a promising approach in the prevention and treatment of CVDs [2]. In the context of different CVDs, mitochondrial protein quality controls systems have attracted wide attention [3,4].

The heart cells have a very high demand for metabolites; thus, the protein turnover, synthesis and quality control are under tight control, especially in mitochondria, sarcomeres and sarcoplasmic reticulum [5]. CVDs are known to be characterised by increased reactive oxygen species (ROS), oxidative and mechanical stresses, leading to the accumulation of misfolded/damaged proteins and mitochondrial DNA mutations [6]. Ageing is a normal physiological process, which is characterised by a general decline in the efficacy of protein quality control systems and disruptions of protein and mitochondria degradation pathways, thus resulting in a wide range of age-associated cardiac dysfunction and a high risk of CVDs development [7]. Molecular chaperones are one of the crucial classes of proteins, involved in heart protection against stresses- and age-mediated accumulation of toxic misfolded proteins by regulation of the protein synthesis/degradation balance and refolding of misfolded proteins [8].

There are four known isoforms of HSP90 chaperone: cytosolic *HSP90a* and *HSP90b*, mitochondrial TNF receptor-associated protein 1 or HSP90N (*TRAP1*) and ER-localised glucose-regulated protein, 94 kDa (*Grp94*). HSP90a and HSP90b are the most abundant molecular chaperones in the cytosol. While *HSP90b* is a constitutively expressed isoform, *HSP90a* is inducible by stress [9]. Three functional domains (N-terminal ATP-binding, medial protein-binding and C-terminal dimerizing domain) are involved in protein stabilisation against heat stress and assist in protein degradation. Upon interaction with misfolded proteins, HSP90 decreases their aggregation with other misfolded proteins, further linking it with protein degradation and destruction (via polyubiquitination and 26S proteasome, respectively) [10]. Currently, for HSP90, several hundreds of substrate and/or client proteins, dozens of co-chaperones (steroid receptors, glucocorticoid receptor, immunophilin co-chaperones and many others) have been identified [11]. Thus, the role of HSP90 in the development and progress of several types of cancer, many neurodegenerative and heart diseases is not surprising. In this review, we focus on the recently investigated role of HSP90 proteins in CVDs development, mechanisms of HSP90s-mediated cardioprotection and discuss molecular pathways and signalling mechanisms related to heart ageing.

## 2. The Role of HSPs in CVDs

The primary activity of HSPs is stimulated by a wide range of stress factors, such as oxidation and heat. This process, named heat shock response (HSR), is a well-conserved protective mechanism, providing cellular protection via proper folding and maturation of proteins, inhibition of caspase-dependent apoptosis and activation of several other signalling pathways [12]. Transcription factor (TF) Heat shock factor 1 (HSF1) is the main regulator of *HSPs* expression. In normal conditions, *HSF1* is repressed; however, upon stress, HSF1 monomer translocates into the nucleus, forms phosphorylated trimers and binds HSEs (elements), thus activating *HSPs* expression. Closing the regulatory circuit, HSP90 binds and silences HSF1, thus making an inducible HSP90/HSF1/HSE cytoprotective pathway, ensuring cellular protection and damage elimination [13].

HSP90-mediated cytoprotection relies on its multiple interaction partners (or clients), providing their correct folding, activation, transportation, stabilisation and timely degradation. Among HSP90 clients are TF and kinases, involved in cell growth, survival, development and differentiation, such as cell division cycle 37 (CDC37), Mitogen-Activated Protein Kinase 1 (MAPK1), Protein Kinase B (Akt) and many others, making HSP90 an essential molecular hub for normal cellular function and a promising target for pharmacological intervention able to affect multiple molecular pathways [14,15]. Natural and synthetic compounds, targeted specific domains and cofactor interactions of HSP90 are widely used to better understand HSP90 functions and search for client proteins. For example, geldanamycin and radicicol target the ATP-binding site in the N-terminal domain; celastrol targets the interface between HSP90 and CDC37, while novobiocin targets the C-terminal domain [16]. Further, inhibitors are divided based on their method of actions: activating or inhibiting HSR, destabilising the HSP90-client complex, causing client release with or without activation, increasing client degradation and others [17].

In the following sections, we focus on the main cardioprotective properties of HSP90s, their involvement in CVDs development and describe the main interaction partners/clients and known signalling mechanisms concerning particular CVDs.

### 2.1. Cardioprotection Mechanisms (UPR, ER Stress and Apoptosis)

**Figure 1:**
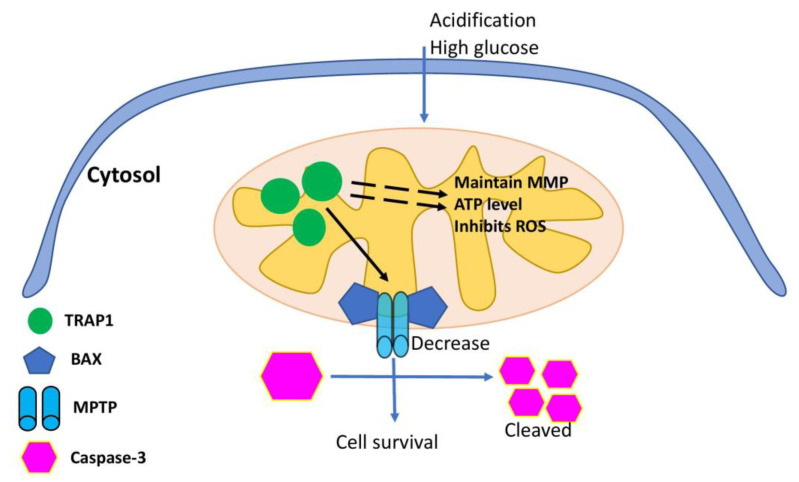
Schematic representation of the TRAP1 mitochondria protective mechanism. TRAP1 protects mitochondria from stress-induced damage, regulates MPTP opening and MMP, maintains normal mitochondrial ultrastructure and cell ATP production and inhibits cell ROS and activation of the mitochondrial apoptotic pathway, thus preventing cell death.

Cardiac microvascular injury is a common diabetes complication, resulting from increased OS and inflammatory responses and induction of unfolded protein response (UPR). While UPR is a mechanism of adaptive response, excessive UPR activation triggers a cascade of pathological events, resulting in apoptosis in specialised tissues and cells. The sensor inositol-requiring enzyme 1 alpha (IRE1α) plays a crucial role in diabetes-mediated microvascular injury, mainly by promoting splicing of X-Box Binding Protein 1 (XBP1), further accelerating the degradation of intracellular Vascular Endothelial Growth Factor A (VEGF) and thus interrupting vascular regeneration (reviewed in [18]). The stability of IRE1α is determined by the association with cytosolic HSP90 chaperone [19], thus linking UPR, HSP90-IRE1α complex and endothelial dysfunction. Additionally, diabetes-mediated hyperglycemia promotes the glycation of proteins and lipids (so-called Advanced glycation end products (AGEs)), which are known to trigger UPR and ER stress, with further induction of apoptosis and impair endothelial integrity and function [20]. Additionally, the protective role on cardiomyocytes from high glucose-mediated injury was shown for both HSP90 (via Akt pathway) and TRAP1 (via regulation of mitochondrial membrane potential (MMP), mitochondrial permeability transition pore (MPTP) opening and ROS level) [21,22,23] (Figure 1).

The additional regulatory pathway was identified on cardiac microvascular endothelial cells culture of the Pellino E3 Ubiquitin Protein Ligase 1 (*PELI1*)-deficient mice model, which is known as an ER stress mediator and regulator of IRE1α stability. The Ring domain of Peli1 directly binds Hsp90 and, subsequently, enhances IRE1α phosphorylation [24]. Thus, prevention or blocking Peli1 interaction with HSP90 may protect against diabetes-mediated cardiac microvascular injury by limiting ER stress.

Recently, the HSP90 inhibitor 17-AAG (tanespimycin) was shown to inhibit ER stress and activation of Nuclear Factor Kappa B (NF-kB) in rat cardiomyocytes, thus preventing apoptosis [25]. The effect of 17-AAG relies on the normal expression of miR-93, an important regulator of cardiomyocytes survival under stress conditions [26]. Similarly, reduction of myocardial cells apoptosis rate was observed on broiler chicken model system, where administration of aspirin leads to upregulation of *HSP90* and *AKT* expression, inhibition of caspase-3 and caspase-9 activities [27]. Confirming results were also obtained on the rats Hsp90 knocked down cardiac microvascular endothelial cells (CMVECs) model, where aspirin treatment stimulates Hsp90 expression, which acts via both Akt and PKM2 (Pyruvate Kinase 2/3) signals to protect CMVECs from the heat-stress damage [28]. Experiments on myocardial tissues of heat-stressed mice also suggested that aspirin administration induce Hsp90 and Akt, stimulate PKM2 signalling, accelerate the mitochondrial translocation of Akt and Pkm2, leading to phosphorylation of mitochondrial BCL2 Apoptosis Regulator (Bcl-2) to protect the integrity of mitochondria from apoptosis initiation [29].

### 2.2. Ischemia/Reperfusion (I/R) Injury

Ischemic conditions, when blood supply is limited or restricted, cause apoptosis of cardiac myocyte and could lead to myocardial cell loss, accelerate cardiac dysfunction and even heart failure. However, effective therapeutic intervention, such as percutaneous coronary angioplasty (PCA), allowing quick restoration of the blood flow, also caused a rise of damaging ROS (called myocardial reperfusion injury (MRI)) and associated with further complications (such as lethal reperfusion, myocardial stunning and reperfusion arrhythmias) [30]. Mitochondrial dysfunction happens early after ischemia when mitochondrial calcium overload and mitochondrial membrane depolarisation lead to the opening of the MPTP and subsequent release of cytochrome c and pro-apoptosis proteins, which could cause even cardiomyocyte death [31].

An effective way to reduce myocardial injury is the application of short and repeated ischemia/reperfusion treatment before reperfusion, which is called Ischemic postconditioning (IpostC) [32]. HSP90 plays a crucial cardioprotective role in IpostC, inhibiting the expression of the complement system (C3 and C5a components), cytokines (*TNFα* and *IL-1β*) and C-Jun N-Terminal Kinase 1 (JNK), thus attenuating I/R-mediated myocardial injury and apoptosis [33]. Similar results were obtained on the rat model of myocardial I/R, where HSP90 reduced expression of inflammation factors (*TNFα*, *IL-1*, *IL-6*, and *ICAM1*), mRNA and expressions levels of the *TLR4* and *NF-kB*-signalling pathways [34]. Additionally, celastrol and celastrol-type HSP90 inhibitors were able to provide protection against hypoxic and I/R stresses via an increase in *HSP70* and *HO-1* genes expression and activation of ERK1/2 and Akt kinases and prevent the opening of the MPTP pore [35]. Similarly, celastrol-type HSP90 inhibitor was shown to reduce the amount of non-viable tissue after ischemia, reduce *HSP70* expression and phosphorylation of ERK and Akt, increase the transcription of the cytoplasmic and mitochondrial inter-membrane located superoxide dismutase (*SOD1*), *SOD2*, catalase (*CAT*), and glutathione-disulfide reductase (*GSR*), thus providing wide-scope cardioprotection [36].

Recently, the central role of mitochondrial HSP90 and connexin 43 (Cx43) in IpostC cardioprotection was shown [37]. Cx43 is the predominant protein forming gap junctions and hemichannels in the ventricular myocardium, thus crucial for intercellular electrical conduction, cardioprotection and cell survival in mammals [38]. Application of IpostC on rat heart-derived H9c2 cells and neonatal rat cardiomyocytes resulted in increased total and mitochondrial levels of HSP90, the mitochondria-to-sarcolemma ratio of Cx43, increased levels of Bcl-2 and decreased Bax levels in both sarcolemma and mitochondrial fractions [37].

### 2.3. Myocardial Fibrosis, Hypertrophy and Heart Failure

Myocardial fibrosis is a hallmark of hypertrophic cardiomyopathy and a proposed substrate for arrhythmias and heart failure. Abnormal accumulation of fibrillar collagen in the extracellular space leads to reduced myocardial stiffness and ventricular dysfunction during cardiac hypertrophy. Cardiac fibroblast is the primary cell type in the myocardium, responsible for the synthesis and secretion of collagen in response to pressure-overload hypertrophy [39]. Several recent reports have proved the involvement of HSP90 in the regulation of the fibrosis process on several layers and via several interaction partners.

Recent research has shown the role of the endothelial cell-specific calpain in myocardiocyte hypertrophy, cell death, myocardial endothelial injury via the HSP90/Akt signalling pathway, thereby promoting cardiac fibrosis [40]. Calpains is a large family of well-known calcium-activated neutral cysteine proteases, playing critical roles in myocardial remodelling [41]. Calpains inhibition or deletion are known to ameliorate myocardial dysfunction, lower the volume of cardiac collagen and endothelial–mesenchymal transition [42]. It was shown that calpain inhibition decreases phosphorylated Akt, and vice versa; activating calpain elevates the level of phosphorylated Akt in mice culture of endothelial cells. Further, upregulation of phosphorylated Akt by calpain promotes the endothelial–mesenchymal transition, whereas inhibition of calpain switches on the protective mechanism during the endothelial–mesenchymal transition via the HSP90/Akt signalling method in endothelial cells [40].

Another way to ameliorate cardiac myocardial fibrosis was discovered via disruption of HSP90/Transforming Growth Factor Beta 1 (TGFβ) chaperon-client association. Application of HSP90 inhibitor CTPR390 prevents binding of HSP90 to its client TGFβ, which resulted in reduced motility of myocardial TGFβ-activated fibroblasts, blocked collagen expression and improved angiotensin-II (AngII) -induced cardiac myocardial fibrosis in vivo in a pro-fibrotic mouse model [43]. Recently, an alternative approach to prevent Hsp90-TGFβ binding was demonstrated. Prevention of Hsp90 S-nitrosylation at cysteine 589 resulted in reduced fibrosis in angiotensin II- or isoproterenol-treated cardiac fibroblasts via blocking the TGFβ/SMAD3 signalling pathway [44]. Similarly, HSP90 inhibitor 17-DMAG (17-dimethylaminoethylamino-17-demethoxygeldanamycin) significantly reversed AngII-induced mitochondrial fission and adventitial fibroblasts to myofibroblasts switch by modulating the calcineurin-dependent dephosphorylation of Dynamin 1 Like (Drp1) [45]. This further supported the close association of traditional pathways of fibroblasts activation (such as TGFβ and AngII) and recently defined mitochondrial and metabolic mechanisms of cardiac fibrosis [46]. Additionally, cardiomyocyte-targeted knockdown of Hsp90 in rats resulted in downregulated collagen synthesis. Mechanically, myocyte-derived Hsp90 regulates interleukin-6 (IL-6) synthesis and its release in exosomal vesicles, which are responsible for the activation of Signal Transducer And Activator Of Transcription 3 (STAT-3) signalling in cardiac fibroblasts and subsequent excess collagen synthesis, leading to severely compromised cardiac function during cardiac hypertrophy [47].

The role of HSP90 in cardiac hypertrophy was further verified under in vivo and in vitro experimental conditions [48]. Application of HSP90 inhibitor 17-AAG prevented cardiac dysfunction and cardiac hypertrophy after myocardial infarction. Mechanically, HSP90 acts via its client Raf1, when HSP90 inhibition leads to ubiquitination and trafficking of Raf1 to the proteasome, thus affecting phosphorylation levels of c-Raf downstream targets—Erk1/2 and GATA4 in cardiomyocytes. Raf1 is a proto-oncogene, serine/threonine kinase, known to activate many downstream targets (such as dual-specificity protein kinases MEK1 and MEK2, which in turn, subsequently activate the serine/threonine-specific protein kinases ERK1 and ERK2), providing pleiotropic effects on cell division cycle, apoptosis, cell differentiation and cell migration [49]. Thus, the pathophysiological role of HSP90 in cardiac hypertrophy relies on the activation of the Raf/Mek/Erk pathway [48].

### 2.4. Atherosclerosis

Atherosclerosis is a chronic inflammatory disease, in which cholesterol accumulates in the arterial wall, leading to the formation of foam cells and plaque, which is a high-risk factor for many complications (depending on which arteries are affected) [50].

HSP90 is the key chaperon of cellular stress response, known to interfere with the inflammatory, cellular and humoral autoimmune responses and pro-oxidative factors in experimental atherosclerosis [51]. HSP90 activates NF-kB to enter the nucleus, promoting inflammatory response and pro-oxidant gene transcription. Multiple studies have found functional crosstalk between NF-kB and nuclear factor erythroid-derived 2-like 2 (Nrf2), the main transcriptional regulator of cellular defence against oxidative stress and electrophiles, and suggested their potential as therapeutic targets [52]. Application of HSP90 inhibitor 17-DMAG induces Nrf2 activation in the aortic tissue which was associated with NF-kB inhibition in atherosclerotic plaques, reduction of lesion size and inflammatory components. Additionally, 17-DMAG atheroprotective activities were linked to the induction of antioxidants (superoxide dismutase, catalase and heme oxygenase-1), autophagy and cytoprotective HSP70 in diabetic mice aortic tissue [53]. In addition to the association with NF-kB, HSP90 up-regulates plaque *MMP-8* (matrix metalloproteinase 8) which is responsible for the degradation of type I, II and III collagens, thus might be the underlying mechanism of the change in plaque vulnerability index [54].

Recently, the role of HSP90 was shown in another atheroinflammatory process–calcific aortic valve disease (CAVD) [55], which is a progressive disease caused by an accumulation of oxidised lipids and the infiltration of inflammatory cells into the valve, resulting in a thickening of the aortic valve leaflets, progressive calcification and ultimately a severe obstruction of cardiac outflow [56]. There are many signalling pathways, transcription factors and proteins that have been linked to CAVD, such as osteoblastic differentiation and skeletal morphogenesis regulating TF Runt-Related Transcription Factor 2 (*RUNX2*), bone morphogenic protein 2 (*BMP2*), several matrix metalloproteinases and interleukins, Tumour Necrosis Factor-Alpha (*TNFα*); however, the exact factors driving the CAVD progression are not known [57]. Recent proteomic investigations have defined an upregulation of Serum Amyloid P-Component (*APCS*), Complement Component C9 or ARMD15 (*C9*) and transgelin (*TAGLN* gene), and a downregulation of *HSP90*, Protein Disulfide Isomerase-Associated 3 (*PDIA3*), Annexin A2 (*ANXA2*) and Beta-Galactoside-Binding Lectin L-14-I or *GBP* (galectin-1) proteins in calcified valves [55]. In general, those results are well-supported by another research. *APCS*, the member of the Pentraxin family, is involved in amyloidosis but it is also known to exert anti-inflammatory and antifibrotic properties (in particular, to inhibit monocyte differentiation into proinflammatory macrophages), thus could be used as inflammation and large artery atherosclerosis biomarker [58]. Similarly, the transgelin, acting via the transgelin–actin complex and modulating the reorganisation of the actin cytoskeleton, is known to participate in the regulation of the blood flows of coronary arteries, and thus is a promising therapeutic target for the treatment of myocardial ischemia [59]. Downregulated components, such as ANXA2 and PDIA3 are also associated with the calcification process and coronary artery disease [60,61,62].

In total, we could conclude that HSPs are playing an indirect role in CVDs and CVDs-related risk factors, acting via their client proteins. Thus, regulation of the local HSPs expression, activity and binding specificity could be a new route in CVDs protection and treatment. However, further studies on different in vivo model systems are required to validate and clarify every described molecular signalling mechanism. The discussed involvement of HSPs in the CVDs are summarised in Table 1.

## 3. Heart Ageing

Advanced age is one of the main risk factors for CVDs, due to the gradual decline in all biological processes and increase in accumulated cellular damage. The protein homeostasis system is critical for maintaining organismal viability and cell function; it relies on several main stress response pathways: HSR, UPR^ER^ [63], UPR^mt^ [64], oxidative stress response (OxSR) [65], ubiquitin–proteasome system and autophagy [66]. Mitochondrial dysfunction, DNA damage and impaired repair, Telomere Dysfunction and ROS are the main stressors in cardiac ageing [67]. Further, we will discuss different cellular pathways (both proven and potential) related to heart ageing, in which HSP90 is involved.

### 3.1. Stabilisation and Upregulation of Anti-Poptotic AKT Protein

AKT provides anti-apoptotic functions with two known mechanisms: inhibition of pro-apoptotic signal transduction (Foxo3A, Bax and Bad) and suppress the apoptosis process via stimulation of mTOR and NF-kB pathways. *AKT* is known to be overexpressed in senescent cells, and Hsp90α helps to maintain an upregulated level of AKT, thus stabilising senescent cells from apoptosis. Application of 17-DMAG HSP90 inhibitor on the Ercc1^−/Δ^ mouse (model of a human progeroid syndrome, which spontaneously develops age-related degenerative diseases and has a maximum lifespan of 7 months) resulted in a significant reduction in a composite score of age-related symptoms including tremor, ataxia, kyphosis, loss of forelimb grip strength, gait disorder, coat condition, dystonia and overall body condition [68]. Anti-apoptotic properties of HSP90 were also covered in Section 2.1.

### 3.2. Protein Kinase C Epsilon

Protein kinase C epsilon (PKCe) is a crucial mediator in the cardioprotection of ischemic preconditioning, which was shown to translocate to mitochondria upon activation [69]. Interestingly, the process of PKCe translocation to mitochondria is age-related and relies on the caveolin-3-dependent mechanism and is regulated via *HSP90* and Translocase of Outer Mitochondrial Membrane 70 (*TOM70*) expression [70]. The level of mitochondrial PKCe and expression of *HSP90* and *TOM70* were significantly decreased in middle-aged rat hearts in comparison to young adult rat hearts. While caveolin-3 is a well-known scaffolding protein, involved in cardioprotection via activation of the sarcolemma repair mechanism and cardiomyocytes [71,72], TOM70 is responsible for the translocation of mitochondrial pre-proteins from the cytosol into the mitochondria in an Hsp90-dependent manner. However, only one of the identified C-terminal MEEVD motifs within dimeric Hsp90 is responsible for contacts with Tom70, thus targeting this motif could be used to modulate Hsp90/Tom70 interaction [73].

### 3.3. AMP-Activated Protein Kinase

AMP-Activated Protein Kinase (AMPK) is a crucial cellular energy metabolism sensor, known to decrease with advanced age, and may contribute to the mitochondria and intracellular lipid metabolism dysfunctions [74]. Similarly, aged mice have decreased levels of PPARG Coactivator 1 Alpha (PGC-1), a mitochondrial biogenesis cofactor, known to protect the heart from ischemic insults as a part of the AMPK/PGC-1α signalling cascade [75]. Decreased expression levels of membrane Solute Carrier Family 2 Member 4 (GLUT4) and HSP90 in aged AMPK-deficient mice suggested their crucial role in age-induced cardiac dysfunction, probably via preservation of mitochondrial functions and ROS generation [76].

### 3.4. Hsf1-Mediated Senescence

Hsf1 is the main TF, inducing HSRs (proliferative, drug resistance and anti-apoptosis effects) during stressed cellular conditions. Hsf1 is closely associated with Hsp90α during both proteotoxic stress response and unstressed cells. Application of Hsp90 inhibitor leads to the disruption of the HSP90 and HSF1 interaction, HSF1 translocation to the nucleus and activation of pro-survival HSR [77]. Additionally, the downregulation of Hsf1 was shown in fibroblasts, where senescence was induced by DNA damaging treatments. Such suppression of Hsf1 activates the p38/NF-kB/SASP pathway, where Senescence-associated Secretory Phenotype (SASP) is a different signalling molecule (such as metalloproteinases, pro-inflammatory factors), thus further promoting senescence in neighbouring cells [78]. Accordingly, HSP90 inhibitors could induce the enhanced activity of Hsf1 and prevent senescence in heart cells. However, this idea requires wide experimental confirmation.

### 3.5. Role of the Hsp90 Isoforms in Senescence

Similarly to the main Hsp90 isoforms, mitochondrial (TRAP1) and endoplasmic (Grp94) isoforms can also play a crucial role in senescence by suppressing apoptosis. *TRAP1* is known to overexpress during mitochondrial stress, especially under increased ROS conditions [79]. The high ROS level leads to activation of cyclophilin D (CypD) protein in the MPTP pore opening, subsequently losing the mitochondrial contents and cell death. TRAP1 binds CypD and prevents MPTP leakage and promote survival of stressed cell [80]. These properties of the TRAP1 were implemented in a search for anti-cancer drugs, such as TRAP1 and Grp94 inhibitors [81,82]. Additionally, TRAP1-negative mice have signs of diminished lifespan, with abnormal tissue generation, dysplasia [83], and cells delivered from such mice with impaired cell proliferation were non-viable [84], thus suggesting that TRAP1 inhibitors have potential as senolytic agents. The endoplasmic isoform of Hsp90 is upregulated in response to UPR^ER^ [85], and in cancer cells, where it is involved in the regulation of cancer cells proliferation [86]. Because signalling pathways, promoting cellular damage and destruction are similar in both senescent and cancer cells, the development of the new inhibitors to target specific Hsp90 isoforms could be a promising direction in both the extension of healthy ageing and as an anti-cancer therapy [84]. However, further experiments are required to prove these hypotheses.

### 3.6. SASP Depletion

Several Hsp90 inhibitors (Ganetespib, TAS-116, AUY-922 and BIIB021) have been shown to reduce the level of SASP (particularly IL-8) in malignant pleural mesothelioma. Subsequently, such SASP depletion affects AKT phosphorylation (via Protein Tyrosine Kinase 2 (PTK2), resulting in apoptosis and cell death [87]. These data suggest an indirect influence of Hsp90 on the SASP pool and could be another point of intervention to prevent cellular senescence. However, an experimental conformation for the described effect of the Hsp90 inhibitors on non-cancer or heart cell lines is still missing.

### 3.7. DNA Damage Response

DNA damage leads to activation of the signalling network of DNA damage response (DDR), which relies on several DNA repair mechanisms (excision repair, homologues recombination and non-homologues end-joining) [88]. Additionally, many DDR responsible genes are known as Hsp90α clients (DNA Polymerase Beta, Ataxia Telangiectasia Mutated, APEX Nuclease 1, BRCA1 DNA Repair Associated, FA Complementation Group A and others) [89]. Thus, Hsp90α promotes senescence by supporting the DDR process via stabilisation of pro-DDR proteomes.

### 3.8. TERT (Telomerase Reverse Transcriptase) Enzyme

**Figure 2:**
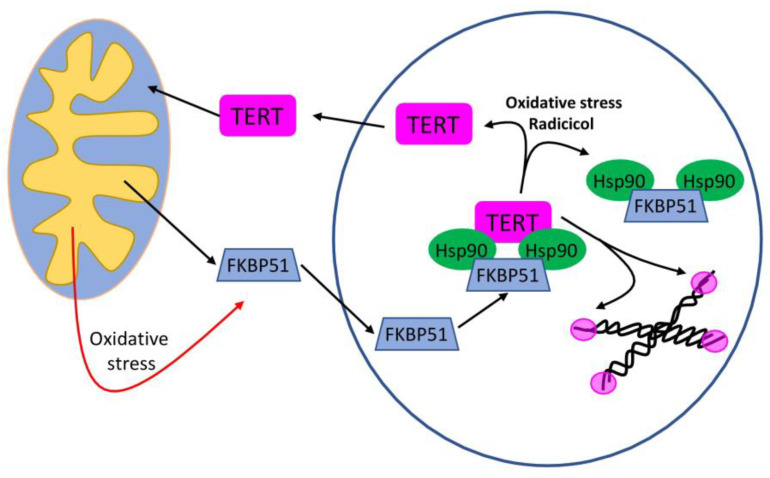
The role of Hsp90 in the regulation of the TERT-immunophilin FKBP51 interaction. TERT is stabilised by the Hsp90-based heterocomplex. TERT is targeted to chromosomes where its telomerase activity prevents chromosome end shortening. Mitochondrial FKBP51 rapidly concentrates in the nucleus by oxidative stress. The TERT exported to mitochondria is facilitated by the chaperoning action of FKBP51 releasing TERT from telomeric regions where it was anchored. Oxidative stress or radicicol disrupt the Hsp90 heterocomplex, promoting TERT nuclear export. Further, the cytoplasmic TERT pool could be imported by mitochondria or degraded by the proteasome. Both FKBP51 and mitochondrial TERT play a protective role against apoptosis.

Telomeres are responsible for chromosomal protection during DNA damage via telomere length shortening, subsequently leading to cellular senescence. A high level of TERT enzyme helps cells to evade senescence due to the ability of the TERT to add a sequence to the cut telomeric end [90]. Hsp90α is known to bind and stabilise TERT coenzyme in cancer cells, thus promoting telomerase activity and suppressing senescence [91]. FKBP51 and FKBP52 are Hsp90-bound immunophilins associated with steroid receptors and proposed as potential modulators of signalling cascade factors chaperoned by Hsp90. The Hsp90 inhibitor radicicol disrupts the heterocomplex with immunophilins. Under oxidative stress, only FKBP51 becomes mostly nuclear, colocalising with hTERT, and telomerase activity of TERT is significantly enhanced by FKBP51, but not FKBP52 [91] (Figure 2). Thus, the application of Hsp90 inhibitor radicicol could be used to enhance telomerase activity.

### 3.9. Senescence Inducer p14ARF

p14ARF protein (encoded by *CDKN2A* gene—Cyclin-Dependent Kinase 4 Inhibitor A) is an important tumour suppressor gene, frequently mutated or deleted in many types of tumours. p14ARF functions as a stabiliser of p53 (tumour suppressor protein) and regulator of CDK4, thus controlling cell cycle G1 progression [92]. The close association between p14ARF and p53 was shown to play a crucial role in the cellular senescence of cancer cells [93]. Additionally, p14ARF binds Mdm2 protein, the proto-oncogenic E3 Ubiquitin-Protein Ligase, known to target tumour suppressor proteins for proteasomal degradation. p14ARF-Mdm2 association prevents Mdm2-mediated ubiquitination, nuclear export and proteasomal p53 degradation [94]. Additionally, the p14ARF-Mdm2 association has p53-independent activity, mediated via regulation of the translation of E2F1 protein, a TF, controlling cell cycle and tumour suppressor proteins [95].

Experimental studies suggested that Hsp90α facilitates lysosomal Lysosomal Associated Membrane Protein 2 (LAMP2A) dependent degradation of p14ARF [96]. Thus, inhibiting the association of Hsp90α with p14ARF will prevent activation of diverse senescence causing signalling pathways. Further, interaction and co-regulation with other proteins (such as p53 and Mdm2) provide additional points for target pathway-specific therapeutic intervention.

### 3.10. Aggregation of Oxidised Proteins

Oxidation stresses of different origins result in the generation of damaged, misfolded, unfolded proteins and proteins with structural abnormalities, the subsequent establishment of cross-links with other oxidised proteins and the formation of insoluble protein aggregates, which greatly favour ageing [97]. The 20S proteasome, which was considered the main system responsible for the degradation of oxidised proteins, requires the assistance of chaperones to transport to the proteasome-oxidised proteins tagged for degradation. Hsp90 is known for its strong binding to actin and ability to enhance actin degradation at the proteasome. However, under oxidative conditions, 20S proteasome function is impaired, and degradation of Hsp90-actin complex resulted in the enhanced deposition of insoluble actin aggregates in cytoplasm and cleaved form of Hsp90 (Hsp90cl). Hsp90cl has no chaperoning function; however, it was shown to acquire a new function in mediating the accumulation of actin aggregates [98]. Thus, decrement in proteasome activity, loss of Hsp90 chaperoning ability and enhanced protein aggregates formation may contribute to the age-related and cell senescence-related cellular events [99].

### 3.11. RAF1 Proto-Oncogene

Proto-Oncogene Serine/Threonine-Protein Kinase (*RAF1*) is involved in the regulation of the cell division cycle, apoptosis, differentiation and migration. As a part of the MAPK cascade, RAF1 was also associated with senescence of skin tissue [100]. Recently, it was shown that Hsp90 chaperoning activity is required for proper RAF oncogene adaptation. Application of Hsp90 inhibitor 17AAG prevents normal Raf1 function and inhibited cell proliferation. Thus, acting as a tumour suppressor, 17AAG triggered premature cellular senescence in tumour cells and deprived their ability to proliferate and metastasise in the mice model system [101].

The primary beneficial functions of HSPs are based on their effect on the regulation of apoptosis and cell cycle progression. In addition to the described cardioprotective and senolytic properties, a high number of recent papers suggest the involvement of HSPs in stress-mediated response, immunomodulation, neurodegenerative diseases and many types of cancer (reviewed in [102]). Thus, the development of HSPs-target agents able to specifically kill or suppress senescent or cancer cells would have significant therapeutic effects to provide healthy ageing, treat age-related diseases and improve resiliency. Indeed, certain HSPs inhibitors are undergoing clinical trials and others will be available for practitioners shortly (reviewed in [4,103,104]).

**Figure 3:**
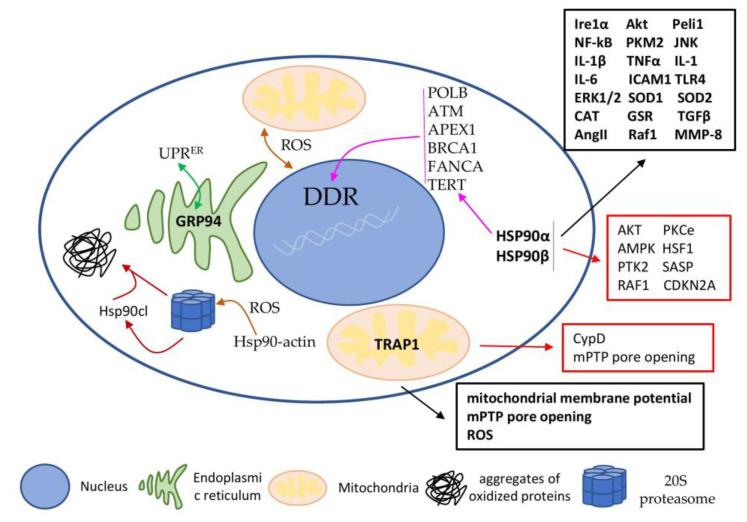
Involvement of HSP90 and its homologues in CVDs and heart senescence. HSP90α and β are the main isoforms localised in the cytoplasm or the nucleus. It can also be transported to the mitochondria, where homologue TRAP1 exists. The ER homologue GRP94 is involved in UPR^ER^ (green line). Through the interaction with different co-chaperones and client proteins, HSPs have engaged in a wide range of important cellular signalling pathways. Black boxes list CVDs-related interaction/client proteins and activated signalling pathways. Red boxes list senescence-related interaction/client proteins and activated signalling pathways. Magenta lines designate DDR-related interaction/client proteins. Brown lines designate involvement of HSP90 in the ROS-mediated accumulation of aggregated proteins and release of the cleaved HSP90 form.

The discussed involvement of HSP90 protein in CVDs and heart ageing have been summarised in Figure 3.

## 4. Cancer, CVDs and Ageing Treatment with HSP90 Inhibitors

Enhanced expression of HSP90 has been reported in many types of cancer and is associated with a more aggressive tumour phenotype. However, HSP90 is expressed in most human cells. Thus, the major concern for HSP90 inhibitors design is high specificity to target cancer cells. Many selective HSP90 inhibitors have been synthesised and used in clinical trials [105,106,107]. A recent study suggests the combined application of TRAP1 inhibitor gamitrnib and pan-HSP90 (inhibits all HSP90 paralogs—HSP90, TRAP1 and GRP94) inhibitor DN401 (purine scaffold small molecule) [108]. Furthermore, many GRP94-specific inhibitors were developed (PU-WS13, PU-H39, and PU-H54), which were 100-fold more selective for GRP94 over HSP90 α/β and were able to induce apoptosis of human breast cancer cells [109]. When administered into tumour-bearing mice, GRP94-specific inhibitor compound 18c was cleared rapidly from normal tissue while being selectively retained in the tumour, where 18c inhibited the post-ER expression of TLR9, and also reduced the steady-state levels of HER2 kinase of breast cancer cells [110]. The main complication to all previous and current HSP90 inhibitors in clinical trials is severe toxicity. Nevertheless, the development of an HSP90-based tumour vaccine, monoclonal antibodies and small molecular inhibitors is another step in the future for successful cancer treatment.

Similar to cancer-targeted HSP90 inhibitors, there are several promising publications suggesting the application of HSP90 inhibitors as senolytic drugs. In particular, 17-AAG (Tanespimycin), Geldanamycin and 17-DMAG (Alvespimycin) have been demonstrated to be effective in reducing senescent cell burden in aged mice and cell cultures [68]. Application of senolytic agents of different types (HSP90 inhibitors, ROS-protective, pro-apoptotic and others) was shown to provide several beneficial (including cardiovascular) effects in old mice: enhanced vascular reactivity, increased coat density, improved cardiac ejection fraction and fractional shortening. Additionally, in a HFD (high-fat diet) LdlR^−/−^ mice reduced senescent cell-like, intimal foam cell/macrophages in vascular plaques, and in hypercholesterolemic HFD ApoE^−/−^ mice decreased vascular calcification and increased vascular reactivity were reported (reviewed in [111]). Cardioprotective and myocardial anti-inflammatory properties were shown for geldanamycin applied on I/R and IPostC rats [34].

Despite promising senolytic properties and cardiovascular benefits, HSP90 inhibitors are toxic, and, when used in combination with other drugs, could lead to the development of unknown side effects. However, promising outcomes of many research and proven efficiency in different types of cell cultures and mouse models suggest the tremendous potential of HSP90-based therapy and the ability to treat age-related diseases and ageing itself. Yet, there is still much to learn about their molecular mechanisms of action, which should lead to safer and more effective medications. 

## 5. Conclusions

In total, there are many reasons to admire the substantial health and economic potential of the new class of HSPs-based drugs. New therapeutic approaches to reduce age-related senescent cell burden specifically in the heart and the entire organism can dramatically improve the current method of treating CVDs and age-related diseases. Currently, the primary way to explore HSPs-related functions relies on the prevention/modification of their interaction with client proteins by application of highly specific inhibitors. However, there are still so many open questions to answer and molecular mechanisms to identify, characterise and apply this knowledge to improve human health.

## Figures and Tables

**Table 1:** Association between HSP90 and CVDs with discovered molecular mechanism of action.

CVDs and CVD Risk Factors	Target	Involved Pathway/Activity	References
Microvascular injury	IRE1α	IRE1α-*XBP1*-VEGF pathway; interrupt vascular regeneration	[18,19]
	Peli1	Peli1 enhances IRE1α phosphorylation	[24]
High glucose-mediated injury	Akt pathway	Regulate of mitochondrial membrane potential and MPTP opening	[21,22,23]
Hypoxia/reoxygenation injury apoptosis	miR-93; NF-kB pathway	NF-kB-mediated prevention of apoptosis	[25,26]
Heat stress-induced apoptosis	Akt pathway	Aspirin inhibits caspase-3 and caspase-9 activities	[27]
	Akt and Pkm2 pathways	Aspirin protects CMVECs from the heat-stress damage	[28,29]
I/R, IpostC and myocardial injury	Complement system, JNK, cytokines	Attenuate I/R-mediated myocardial injury and apoptosis	[33]
	TLR4 and NF-kB pathways	Reduced expression of *TNFα*, *IL-1*, *IL-6* and *ICAM1*, and *TLR4* and *NF-kB*-signalling pathways	[34]
	Akt pathway	Protects against hypoxic and I/R stresses	[35]
	Antioxidants	Celastrol-type HSP90 inhibitor increases the transcription of SOD1, SOD2, CAT, and GSR	[36]
	Cx43	Cardioprotection, reduction of redox stress	[37]
	Akt pathway	Inhibits calpain in ECs, alleviates cardiac remodelling and fibrosis in mice	[40]
Myocardial fibrosis	TGFβ	HSP90 inhibitor CTPR390 reduces motility of myocardial TGFβ-activated fibroblasts, blocks collagen expression and improves AngII-induced cardiac myocardial fibrosis	[43]
	TGFβ/SMAD3 pathway	Reduced fibrosis	[44]
	calcineurin/Drp1 pathways	HSP90 inhibitor 17-DMAG suppresses adventitial fibroblasts transformation and adventitial remodelling in hypertensive mice	[45]
	STAT-3	Hsp90 regulates collagen biosynthesis in fibroblasts during cardiac hypertrophy and associated fibrosis	[47]
Cardiac hypertrophy	Raf/Mek/Erk pathway	Hsp90 involved in the development of cardiac hypertrophy following myocardial infarction	[48]
Atherosclerosis	NF-kB pathway	HSP90 promotes inflammatory response and pro-oxidant gene transcription	[52]
	NF-kB pathway	HSP90 inhibitor 17-DMAG reduces lesion size and inflammatory components in atherosclerotic plaques	[53]
	MMP-8; NF-kB pathway	HSP90 regulates plaque development, vulnerability and inflammation	[54]
	Akt/Erk/p38 pathways	HSP90 participates in the progression of aortic valve calcification	[55]

## Abbreviations

AGEs	Advanced glycation end products
AKT	Protein Kinase B
AngII	Angiotensin-II
ANXA2	Annexin A2
APCS	Serum Amyloid P-Component
Bcl-2	BCL2 Apoptosis Regulator
BMP2	Bone morphogenic protein 2
C9	Complement Component C9 or ARMD15
CAT	Catalase
CDC37	Cell division cycle 37
CDKN2A	Cyclin-Dependent Kinase 4 Inhibitor A
CVDs	Cardiovascular diseases
Cx43	Connexin 43
CypD	Cyclophilin D
DDR	DNA damage response
galectin-1	Beta-Galactoside-Binding Lectin L-14-I or GBP
GLUT4	Solute Carrier Family 2 Member 4
Grp94	Glucose-regulated protein, 94 kDa
GSR	Glutathione-disulfide reductase
HSEs	Heat shock elements
HSF1	Heat shock factor 1
HSR	Heat shock response
IpostC	Ischemic postconditioning
IRE1α	Inositol-requiring enzyme 1 alpha
JNK	C-Jun N-Terminal Kinase 1
LAMP2A	Lysosomal Associated Membrane Protein 2
MAPK1	Mitogen-Activated Protein Kinase 1
MPTP	Mitochondrial permeability transition pore
MRI	Myocardial reperfusion injury
Nrf2	Nuclear factor erythroid-derived 2-like 2
PCA	Percutaneous coronary angioplasty
PDIA3	Protein Disulfide Isomerase-Associated 3
PELI1	Pellino E3 Ubiquitin Protein Ligase 1
PGC-1	PPARG Coactivator 1 Alpha
PKCe	Protein kinase C epsilon
PKM2	Pyruvate Kinase 2
PTK2	Protein Tyrosine Kinase 2
RAF1	Proto-Oncogene Serine/Threonine-Protein Kinase
RUNX2	Runt-Related Transcription Factor 2
SASP	Senescence-associated Secretory Phenotype
SOD1	Superoxide dismutase
STAT-3	Signal Transducer And Activator Of Transcription 3
TF	Transcription factor
TGFβ	Transforming Growth Factor Beta 1
TNFα	Tumour Necrosis Factor-Alpha
TOM70	Translocase of Outer Mitochondrial Membrane 70
transgelin	TAGLN gene
TRAP1	TNF receptor-associated protein 1 or HSP90N
UPR	Unfolded protein response)
VEGF	Vascular Endothelial Growth Factor A
XBP1	X-Box Binding Protein 1

## References

[1] WHO CVDs Fact Sheets. Cardiovascular Diseases (CVDs). https://www.who.int/news-room/fact-sheets/detail/cardiovascular-diseases-(cvds).

[2] Tian R., Colucci W.S., Arany Z., Bachschmid M.M., Ballinger S.W., Boudina S., Bruce J.E., Busija D.W., Dikalov S., Dorn G.W. (2019). Unlocking the Secrets of Mitochondria in the Cardiovascular System: Path to a Cure in Heart Failure—A Report from the 2018 National Heart, Lung, and Blood Institute Workshop. Circulation.

[3] Ranek M.J., Stachowski M.J., Kirk J.A., Willis M.S. (2018). The Role of Heat Shock Proteins and Co-Chaperones in Heart Failure. Phil. Trans. R. Soc. B.

[4] Der Sarkissian S., Aceros H., Williams P., Scalabrini C., Borie M., Noiseux N. (2020). Heat Shock Protein 90 Inhibition and Multi-target Approach to Maximize Cardioprotection in Ischaemic Injury. Br. J. Pharmacol..

[5] Carlisle C., Prill K., Pilgrim D. (2017). Chaperones and the Proteasome System: Regulating the Construction and Demolition of Striated Muscle. IJMS.

[6] Quiles J.M., Gustafsson Å.B. (2020). Mitochondrial Quality Control and Cellular Proteostasis: Two Sides of the Same Coin. Front. Physiol..

[7] Ghosh R., Vinod V., Symons J.D., Boudina S. (2020). Protein and Mitochondria Quality Control Mechanisms and Cardiac Aging. Cells.

[8] Mishra S., Dunkerly-Eyring B.L., Keceli G., Ranek M.J. (2020). Phosphorylation Modifications Regulating Cardiac Protein Quality Control Mechanisms. Front. Physiol..

[9] Biebl M.M., Buchner J. (2019). Structure, Function, and Regulation of the Hsp90 Machinery. Cold Spring Harb. Perspect. Biol..

[10] Hoter A., El-Sabban M., Naim H. (2018). The HSP90 Family: Structure, Regulation, Function, and Implications in Health and Disease. IJMS.

[11] Didier Picard P. Lab Hsp90 Interactors. https://www.picard.ch/downloads/Hsp90interactors.pdf.

[12] Lang B.J., Guerrero M.E., Prince T.L., Okusha Y., Bonorino C., Calderwood S.K. (2021). The Functions and Regulation of Heat Shock Proteins; Key Orchestrators of Proteostasis and the Heat Shock Response. Arch. Toxicol..

[13] Masser A.E., Ciccarelli M., Andréasson C. (2020). Hsf1 on a Leash—Controlling the Heat Shock Response by Chaperone Titration. Exp. Cell Res..

[14] Rizzolo K., Houry W.A. (2019). Multiple Functionalities of Molecular Chaperones Revealed through Systematic Mapping of Their Interaction Networks. J. Biol. Chem..

[15] Streicher J.M. (2019). The Role of Heat Shock Proteins in Regulating Receptor Signal Transduction. Mol. Pharmacol..

[16] Banerjee M., Hatial I., Keegan B.M., Blagg B.S.J. (2021). Assay Design and Development Strategies for Finding Hsp90 Inhibitors and Their Role in Human Diseases. Pharmacol. Ther..

[17] Serwetnyk M.A., Blagg B.S.J. (2021). The Disruption of Protein−protein Interactions with Co-Chaperones and Client Substrates as a Strategy towards Hsp90 Inhibition. Acta Pharm. Sin. B.

[18] Gong J., Wang X., Wang T., Chen J., Xie X., Hu H., Yu F., Liu H., Jiang X., Fan H. (2017). Molecular Signal Networks and Regulating Mechanisms of the Unfolded Protein Response. J. Zhejiang Univ. Sci. B.

[19] Datta Chaudhuri R., Banerjee D., Banik A., Sarkar S. (2020). Severity and Duration of Hypoxic Stress Differentially Regulates HIF-1α-Mediated Cardiomyocyte Apoptotic Signalling Milieu during Myocardial Infarction. Arch. Biochem. Biophys..

[20] Liu Z., Zhu H., Ma Y., Tang Z., Zhao N., Wang Y., Pan S. (2021). AGEs Exacerbates Coronary Microvascular Dysfunction in NoCAD by Activating Endoplasmic Reticulum Stress-Mediated PERK Signalling Pathway. Metabolism.

[21] Ke X., Chen J., Peng L., Zhang W., Yang Y., Liao X., Mo L., Guo R., Feng J., Hu C. (2017). Heat Shock Protein 90/Akt Pathway Participates in the Cardioprotective Effect of Exogenous Hydrogen Sulfide against High Glucose-Induced Injury to H9c2 Cells. Int. J. Mol. Med..

[22] Zhang X., Zhong Z., Li W. (2019). Downregulation of TRAP1 Aggravates Injury of H9c2 Cardiomyocytes in a Hyperglycemic State. Exp. Ther. Med..

[23] Zhang L., Liu L., Li X., Zhang X., Zhao J., Luo Y., Guo X., Zhao T. (2020). TRAP1 Attenuates H9C2 Myocardial Cell Injury Induced by Extracellular Acidification via the Inhibition of MPTP Opening. Int. J. Mol. Med..

[24] Zhao Q., Yang J., Chen H., Li J., Que L., Zhu G., Liu L., Ha T., Chen Q., Li C. (2019). Peli1 Induction Impairs Cardiac Microvascular Endothelium through Hsp90 Dissociation from IRE1α. Biochim. Biophys. Acta (BBA)-Mol. Basis Dis..

[25] Guo J., Li S., Li Y., Yan C., Wan Q., Wang Z. (2019). HSP90 Inhibitor 17-AAG Prevents Apoptosis of Cardiomyocytes via MiR-93–Dependent Mitigation of Endoplasmic Reticulum Stress. J. Cell. Biochem..

[26] Yan L.-J., Fan X.-W., Yang H.-T., Wu J.-T., Wang S.-L., Qiu C.-G. (2017). MiR-93 Inhibition Ameliorates OGD/R Induced Cardiomyocyte Apoptosis by Targeting Nrf2. Eur. Rev. Med. Pharmacol. Sci..

[27] Zhang X., Zhang M., Su Y., Wang Z., Zhao Q., Zhu H., Qian Z., Xu J., Tang S., Wu D. (2018). Inhibition of Heat Stress-Related Apoptosis of Chicken Myocardial Cells through Inducing Hsp90 Expression by Aspirin Administration in Vivo. Br. Poult. Sci..

[28] Zhang X., Chen B., Wu J., Sha J., Yang B., Zhu J., Sun J., Hartung J., Bao E. (2020). Aspirin Enhances the Protection of Hsp90 from Heat-Stressed Injury in Cardiac Microvascular Endothelial Cells Through PI3K-Akt and PKM2 Pathways. Cells.

[29] Zhang X., Wu J., Sha J., Yang B., Sun J., Bao E. (2020). Heat Shock Protein 90 Relieves Heat Stress Damage of Myocardial Cells by Regulating Akt and PKM2 Signalling In vivo. Int. J. Mol. Med..

[30] González-Montero J., Brito R., Gajardo A.I., Rodrigo R. (2018). Myocardial Reperfusion Injury and Oxidative Stress: Therapeutic Opportunities. WJC.

[31] Qin C., Wu X., Gu J., Du D., Guo Y. (2020). Mitochondrial Dysfunction Secondary to Endoplasmic Reticulum Stress in Acute Myocardial Ischemic Injury in Rats. Med. Sci. Monit..

[32] Khan H., Kashyap A., Kaur A., Singh T.G. (2020). Pharmacological Postconditioning: A Molecular Aspect in Ischemic Injury. J. Pharm. Pharmacol..

[33] Wang D.-X., Huang Z., Li Q.-J., Zhong G.-Q., He Y., Huang W.-Q., Cao X.-L., Tu R.-H., Meng J.-J. (2020). Involvement of HSP90 in Ischemic Postconditioning-Induced Cardioprotection by Inhibition of the Complement System, JNK and Inflammation. Acta Cir. Bras..

[34] Zhang X.-Y., Huang Z., Li Q.-J., Zhong G.-Q., Meng J.-J., Wang D.-X., Tu R.-H., Wu H.-W. (2020). Role of HSP90 in Suppressing TLR4-Mediated Inflammation in Ischemic Postconditioning. Clin. Hemorheol. Microcirc..

[35] Aceros H., Der Sarkissian S., Borie M., Stevens L.-M., Mansour S., Noiseux N. (2019). Celastrol-Type HSP90 Modulators Allow for Potent Cardioprotective Effects. Life Sci..

[36] Aceros H., Der Sarkissian S., Borie M., Pinto Ribeiro R.V., Maltais S., Stevens L.-M., Noiseux N. (2020). Novel Heat Shock Protein 90 Inhibitor Improves Cardiac Recovery in a Rodent Model of Donation after Circulatory Death. J. Thorac. Cardiovasc. Surg..

[37] Tu R.-H., Li Q.-J., Huang Z., He Y., Meng J.-J., Zheng H.-L., Zeng Z.-Y., Zhong G.-Q. (2017). Novel Functional Role of Heat Shock Protein 90 in Mitochondrial Connexin 43-Mediated Hypoxic Postconditioning. Cell. Physiol. Biochem..

[38] Hirschhäuser C., Lissoni A., Görge P.M., Lampe P.D., Heger J., Schlüter K.-D., Leybaert L., Schulz R., Boengler K. (2021). Connexin 43 Phosphorylation by Casein Kinase 1 Is Essential for the Cardioprotection by Ischemic Preconditioning. Basic Res. Cardiol..

[39] Meagher P.B., Lee X.A., Lee J., Visram A., Friedberg M.K., Connelly K.A. (2021). Cardiac Fibrosis: Key Role of Integrins in Cardiac Homeostasis and Remodeling. Cells.

[40] Sun X., Sun Y., Jiang P., Qi G., Chen X. (2020). Crosstalk between Endothelial Cell-Specific Calpain Inhibition and the Endothelial-Mesenchymal Transition via the HSP90/Akt Signalling Pathway. Biomed. Pharmacother..

[41] Zhang Y., Su S., Li W., Ma Y., Shen J., Wang Y., Shen Y., Chen J., Ji Y., Xie Y. (2021). Piezo1-Mediated Mechanotransduction Promotes Cardiac Hypertrophy by Impairing Calcium Homeostasis to Activate Calpain/Calcineurin Signalling. Hypertension.

[42] Potz B.A., Sabe A.A., Sabe S.A., Lawandy I.J., Abid M.R., Clements R.T., Sellke F.W. (2020). Calpain Inhibition Decreases Myocardial Fibrosis in Chronically Ischemic Hypercholesterolemic Swine. J. Thorac. Cardiovasc. Surg..

[43] Cáceres R.A., Chavez T., Maestro D., Palanca A.R., Bolado P., Madrazo F., Aires A., Cortajarena A.L., Villar A.V. (2018). Reduction of Cardiac TGFβ-Mediated Profibrotic Events by Inhibition of Hsp90 with Engineered Protein. J. Mol. Cell. Cardiol..

[44] Zhang X., Zhang Y., Miao Q., Shi Z., Hu L., Liu S., Gao J., Zhao S., Chen H., Huang Z. (2021). Inhibition of HSP90 S-nitrosylation Alleviates Cardiac Fibrosis via TGFβ/SMAD3 Signalling Pathway. Br. J. Pharmacol..

[45] Huang G., Cong Z., Wang X., Yuan Y., Xu R., Lu Z., Wang X., Qi J. (2020). Targeting HSP90 Attenuates Angiotensin II-Induced Adventitial Remodelling via Suppression of Mitochondrial Fission. Cardiovasc. Res..

[46] Gibb A.A., Lazaropoulos M.P., Elrod J.W. (2020). Myofibroblasts and Fibrosis: Mitochondrial and Metabolic Control of Cellular Differentiation. Circ. Res..

[47] Datta R., Bansal T., Rana S., Datta K., Datta Chaudhuri R., Chawla-Sarkar M., Sarkar S. (2017). Myocyte-Derived Hsp90 Modulates Collagen Upregulation via Biphasic Activation of STAT-3 in Fibroblasts during Cardiac Hypertrophy. Mol. Cell. Biol..

[48] Tamura S., Marunouchi T., Tanonaka K. (2019). Heat-Shock Protein 90 Modulates Cardiac Ventricular Hypertrophy via Activation of MAPK Pathway. J. Mol. Cell. Cardiol..

[49] Parate S., Rampogu S., Lee G., Hong J.C., Lee K.W. (2021). Exploring the Binding Interaction of Raf Kinase Inhibitory Protein With the N-Terminal of C-Raf Through Molecular Docking and Molecular Dynamics Simulation. Front. Mol. Biosci..

[50] Zhu Y., Xian X., Wang Z., Bi Y., Chen Q., Han X., Tang D., Chen R. (2018). Research Progress on the Relationship between Atherosclerosis and Inflammation. Biomolecules.

[51] Profumo E., Buttari B., Tinaburri L., D’Arcangelo D., Sorice M., Capozzi A., Garofalo T., Facchiano A., Businaro R., Kumar P. (2018). Oxidative Stress Induces HSP90 Upregulation on the Surface of Primary Human Endothelial Cells: Role of the Antioxidant 7,8-Dihydroxy-4-Methylcoumarin in Preventing HSP90 Exposure to the Immune System. Oxidative Med. Cell. Longev..

[52] Krajka-Kuźniak V., Baer-Dubowska W. (2021). Modulation of Nrf2 and NF-ΚB Signalling Pathways by Naturally Occurring Compounds in Relation to Cancer Prevention and Therapy. Are Combinations Better Than Single Compounds?. IJMS.

[53] Lazaro I., Oguiza A., Recio C., Lopez-Sanz L., Bernal S., Egido J., Gomez-Guerrero C. (2017). Interplay between HSP90 and Nrf2 Pathways in Diabetes-Associated Atherosclerosis. Clínica Investig. Arterioscler..

[54] Mu H., Wang L., Zhao L. (2017). HSP90 Inhibition Suppresses Inflammatory Response and Reduces Carotid Atherosclerotic Plaque Formation in ApoE Mice. Cardiovasc. Ther..

[55] Weisell J., Ohukainen P., Näpänkangas J., Ohlmeier S., Bergmann U., Peltonen T., Taskinen P., Ruskoaho H., Rysä J. (2019). Heat Shock Protein 90 Is Downregulated in Calcific Aortic Valve Disease. BMC Cardiovasc. Disord.

[56] Tsimikas S. (2019). Potential Causality and Emerging Medical Therapies for Lipoprotein(a) and Its Associated Oxidised Phospholipids in Calcific Aortic Valve Stenosis. Circ. Res..

[57] Conte M., Petraglia L., Campana P., Gerundo G., Caruso A., Grimaldi M.G., Russo V., Attena E., Leosco D., Parisi V. (2021). The Role of Inflammation and Metabolic Risk Factors in the Pathogenesis of Calcific Aortic Valve Stenosis. Aging Clin. Exp. Res..

[58] Zhang Y., Hu H., Liu C., Wu J., Zhou S., Zhao T. (2021). Serum Pentraxin 3 as a Biomarker for Prognosis of Acute Minor Stroke Due to Large Artery Atherosclerosis. Brain Behav..

[59] Zhong W., Sun B., Gao W., Qin Y., Zhang H., Huai L., Tang Y., Liang Y., He L., Zhang X. (2018). Salvianolic Acid A Targeting the Transgelin-Actin Complex to Enhance Vasoconstriction. EBioMedicine.

[60] Cui L., Rashdan N.A., Zhu D., Milne E.M., Ajuh P., Milne G., Helfrich M.H., Lim K., Prasad S., Lerman D.A. (2017). End Stage Renal Disease-Induced Hypercalcemia May Promote Aortic Valve Calcification via Annexin VI Enrichment of Valve Interstitial Cell Derived-Matrix Vesicles. J. Cell. Physiol..

[61] Maguire P.B., Parsons M.E., Szklanna P.B., Zdanyte M., Münzer P., Chatterjee M., Wynne K., Rath D., Comer S.P., Hayden M. (2020). Comparative Platelet Releasate Proteomic Profiling of Acute Coronary Syndrome versus Stable Coronary Artery Disease. Front. Cardiovasc. Med..

[62] Myasoedova V.A., Di Minno A., Songia P., Massaiu I., Alfieri V., Valerio V., Moschetta D., Andreini D., Alamanni F., Pepi M. (2020). Sex-Specific Differences in Age-Related Aortic Valve Calcium Load: A Systematic Review and Meta-Analysis. Ageing Res. Rev..

[63] Estébanez B., de Paz J.A., Cuevas M.J., González-Gallego J. (2018). Endoplasmic Reticulum Unfolded Protein Response, Aging and Exercise: An Update. Front. Physiol..

[64] Shpilka T., Haynes C.M. (2018). The Mitochondrial UPR: Mechanisms, Physiological Functions and Implications in Ageing. Nat. Rev. Mol. Cell Biol..

[65] Liguori I., Russo G., Curcio F., Bulli G., Aran L., Della-Morte D., Gargiulo G., Testa G., Cacciatore F., Bonaduce D. (2018). Oxidative Stress, Aging, and Diseases. CIA.

[66] Sun-Wang J.L., Ivanova S., Zorzano A. (2020). The Dialogue between the Ubiquitin-Proteasome System and Autophagy: Implications in Ageing. Ageing Res. Rev..

[67] Anderson R., Richardson G.D., Passos J.F. (2018). Mechanisms Driving the Ageing Heart. Exp. Gerontol..

[68] Fuhrmann-Stroissnigg H., Ling Y.Y., Zhao J., McGowan S.J., Zhu Y., Brooks R.W., Grassi D., Gregg S.Q., Stripay J.L., Dorronsoro A. (2017). Identification of HSP90 Inhibitors as a Novel Class of Senolytics. Nat. Commun..

[69] Xu J., Khoury N., Jackson C.W., Escobar I., Stegelmann S.D., Dave K.R., Perez-Pinzon M.A. (2020). Ischemic Neuroprotectant PKCε Restores Mitochondrial Glutamate Oxaloacetate Transaminase in the Neuronal NADH Shuttle after Ischemic Injury. Transl. Stroke Res..

[70] Kang C., Qin J., Osei W., Hu K. (2017). Regulation of Protein Kinase C-Epsilon and Its Age-Dependence. Biochem. Biophys. Res. Commun..

[71] Kong C.H.T., Bryant S.M., Watson J.J., Roth D.M., Patel H.H., Cannell M.B., James A.F., Orchard C.H. (2019). Cardiac-specific Overexpression of Caveolin-3 Preserves T-tubular I Ca during Heart Failure in Mice. Exp. Physiol..

[72] Deng F., Wang S., Zhang L., Xie X., Cai S., Li H., Xie G., Miao H.-L., Yang C., Liu X. (2017). Propofol Through Upregulating Caveolin-3 Attenuates Post-Hypoxic Mitochondrial Damage and Cell Death in H9C2 Cardiomyocytes During Hyperglycemia. Cell. Physiol. Biochem..

[73] Zanphorlin L.M., Lima T.B., Wong M.J., Balbuena T.S., Minetti C.A.S.A., Remeta D.P., Young J.C., Barbosa L.R.S., Gozzo F.C., Ramos C.H.I. (2016). Heat Shock Protein 90 KDa (Hsp90) Has a Second Functional Interaction Site with the Mitochondrial Import Receptor Tom70. J. Biol. Chem..

[74] Inata Y., Piraino G., Hake P.W., O’Connor M., Lahni P., Wolfe V., Schulte C., Moore V., James J.M., Zingarelli B. (2018). Age-Dependent Cardiac Function during Experimental Sepsis: Effect of Pharmacological Activation of AMP-Activated Protein Kinase by AICAR. Am. J. Physiol.-Heart Circ. Physiol..

[75] Quan N., Wang L., Chen X., Luckett C., Cates C., Rousselle T., Zheng Y., Li J. (2018). Sestrin2 Prevents Age-Related Intolerance to Post Myocardial Infarction via AMPK/PGC-1α Pathway. J. Mol. Cell. Cardiol..

[76] Turdi S., Fan X., Li J., Zhao J., Huff A.F., Du M., Ren J. (2010). AMP-Activated Protein Kinase Deficiency Exacerbates Aging-Induced Myocardial Contractile Dysfunction: AMPK Deficiency and Aging. Aging Cell.

[77] Pesonen L., Svartsjö S., Bäck V., de Thonel A., Mezger V., Sabéran-Djoneidi D., Roos-Mattjus P. (2021). Gambogic Acid and Gambogenic Acid Induce a Thiol-Dependent Heat Shock Response and Disrupt the Interaction between HSP90 and HSF1 or HSF2. Cell Stress Chaperones.

[78] Kim G., Meriin A.B., Gabai V.L., Christians E., Benjamin I., Wilson A., Wolozin B., Sherman M.Y. (2012). The Heat Shock Transcription Factor Hsf1 Is Downregulated in DNA Damage-Associated Senescence, Contributing to the Maintenance of Senescence Phenotype: Role of Hsf1 in Senescence. Aging Cell.

[79] Elnatan D., Agard D.A. (2018). Calcium Binding to a Remote Site Can Replace Magnesium as Cofactor for Mitochondrial Hsp90 (TRAP1) ATPase Activity. J. Biol. Chem..

[80] Lebedev I., Nemajerova A., Foda Z.H., Kornaj M., Tong M., Moll U.M., Seeliger M.A. (2016). A Novel In Vitro CypD-Mediated P53 Aggregation Assay Suggests a Model for Mitochondrial Permeability Transition by Chaperone Systems. J. Mol. Biol..

[81] Masgras I., Sanchez-Martin C., Colombo G., Rasola A. (2017). The Chaperone TRAP1 as a Modulator of the Mitochondrial Adaptations in Cancer Cells. Front. Oncol..

[82] Yang S., Yoon N.G., Kim D., Park E., Kim S.-Y., Lee J.H., Lee C., Kang B.H., Kang S. (2021). Design and Synthesis of TRAP1 Selective Inhibitors: H-Bonding with Asn171 Residue in TRAP1 Increases Paralog Selectivity. ACS Med. Chem. Lett..

[83] Im C.-N. (2016). Past, Present, and Emerging Roles of Mitochondrial Heat Shock Protein TRAP1 in the Metabolism and Regulation of Cancer Stem Cells. Cell Stress Chaperones.

[84] Fuhrmann-Stroissnigg H., Niedernhofer L.J., Robbins P.D. (2018). Hsp90 Inhibitors as Senolytic Drugs to Extend Healthy Aging. Cell Cycle.

[85] Kroeger H., Chiang W.-C., Felden J., Nguyen A., Lin J.H. (2019). ER Stress and Unfolded Protein Response in Ocular Health and Disease. FEBS J..

[86] Duan X., Iwanowycz S., Ngoi S., Hill M., Zhao Q., Liu B. (2021). Molecular Chaperone GRP94/GP96 in Cancers: Oncogenesis and Therapeutic Target. Front. Oncol..

[87] Di Martino S., Amoreo C.A., Nuvoli B., Galati R., Strano S., Facciolo F., Alessandrini G., Pass H.I., Ciliberto G., Blandino G. (2018). HSP90 Inhibition Alters the Chemotherapy-Driven Rearrangement of the Oncogenic Secretome. Oncogene.

[88] Yoshida K., Fujita M. (2021). DNA Damage Responses That Enhance Resilience to Replication Stress. Cell. Mol. Life Sci..

[89] Sottile M.L., Nadin S.B. (2018). Heat Shock Proteins and DNA Repair Mechanisms: An Updated Overview. Cell Stress Chaperones.

[90] Nalobin D., Alipkina S., Gaidamaka A., Glukhov A., Khuchua Z. (2020). Telomeres and Telomerase in Heart Ontogenesis, Aging and Regeneration. Cells.

[91] Lagadari M., Zgajnar N.R., Gallo L.I., Galigniana M.D. (2016). Hsp90-Binding Immunophilin FKBP51 Forms Complexes with HTERT Enhancing Telomerase Activity. Mol. Oncol..

[92] Sanaei M., Kavoosi F., Ghasemzadeh V. (2021). Investigation of the Effect of 5-Aza-2’-Deoxycytidine in Comparison to and in Combination with Trichostatin A on P16INK4a, P14ARF, P15INK4b Gene Expression, Cell Growth Inhibition and Apoptosis Induction in Colon Cancer Caco-2 Cell Line. Int. J. Prev. Med..

[93] Wang H., Xu G., Huang Z., Li W., Cai H., Zhang Y., Xiong D., Liu G., Wang S., Xue Z. (2017). NLRP6 Targeting Suppresses Gastric Tumorigenesis via P14 ARF –Mdm2–P53-Dependent Cellular Senescence. Oncotarget.

[94] Nguyen T.T.T., Shingyoji M., Hanazono M., Zhong B., Morinaga T., Tada Y., Shimada H., Hiroshima K., Tagawa M. (2021). An MDM2 Inhibitor Achieves Synergistic Cytotoxic Effects with Adenoviruses Lacking E1B55kDa Gene on Mesothelioma with the Wild-Type P53 through Augmenting NFI Expression. Cell Death Dis..

[95] Gnanasundram S.V., Malbert-Colas L., Chen S., Fusée L., Daskalogianni C., Muller P., Salomao N., Fåhraeus R. (2020). MDM2’s Dual MRNA Binding Domains Co-Ordinate Its Oncogenic and Tumour Suppressor Activities. Nucleic Acids Res..

[96] Han S.Y., Ko A., Kitano H., Choi C.H., Lee M.-S., Seo J., Fukuoka J., Kim S.-Y., Hewitt S.M., Chung J.-Y. (2017). Molecular Chaperone HSP90 Is Necessary to Prevent Cellular Senescence via Lysosomal Degradation of P14ARF. Cancer Res..

[97] Nowotny K., Jung T., Grune T., Höhn A. (2014). Accumulation of Modified Proteins and Aggregate Formation in Aging. Exp. Gerontol..

[98] Castro J.P., Fernando R., Reeg S., Meinl W., Almeida H., Grune T. (2019). Non-Enzymatic Cleavage of Hsp90 by Oxidative Stress Leads to Actin Aggregate Formation: A Novel Gain-of-Function Mechanism. Redox Biol..

[99] Höhn A., Weber D., Jung T., Ott C., Hugo M., Kochlik B., Kehm R., König J., Grune T., Castro J.P. (2017). Happily (n)Ever after: Aging in the Context of Oxidative Stress, Proteostasis Loss and Cellular Senescence. Redox Biol..

[100] De Araújo R., Lôbo M., Trindade K., Silva D.F., Pereira N. (2019). Fibroblast Growth Factors: A Controlling Mechanism of Skin Aging. Ski. Pharm. Physiol..

[101] Kanugovi Vijayavittal A., Amere Subbarao S. (2021). The Conformation-Specific Hsp90 Inhibition Interferes with the Oncogenic RAF Kinase Adaptation and Triggers Premature Cellular Senescence, Hence, Acts as a Tumor Suppressor Mechanism. Biochim. Biophys. Acta (BBA)-Mol. Cell Res..

[102] Shan Q., Ma F., Wei J., Li H., Ma H., Sun P. (2020). Physiological Functions of Heat Shock Proteins. CPPS.

[103] Dutta Gupta S., Bommaka M.K., Banerjee A. (2019). Inhibiting Protein-Protein Interactions of Hsp90 as a Novel Approach for Targeting Cancer. Eur. J. Med. Chem..

[104] Margulis B., Tsimokha A., Zubova S., Guzhova I. (2020). Molecular Chaperones and Proteolytic Machineries Regulate Protein Homeostasis in Aging Cells. Cells.

[105] Doi T., Kurokawa Y., Sawaki A., Komatsu Y., Ozaka M., Takahashi T., Naito Y., Ohkubo S., Nishida T. (2019). Efficacy and Safety of TAS-116, an Oral Inhibitor of Heat Shock Protein 90, in Patients with Metastatic or Unresectable Gastrointestinal Stromal Tumour Refractory to Imatinib, Sunitinib and Regorafenib: A Phase II, Single-Arm Trial. Eur. J. Cancer.

[106] Shimomura A., Yamamoto N., Kondo S., Fujiwara Y., Suzuki S., Yanagitani N., Horiike A., Kitazono S., Ohyanagi F., Doi T. (2019). First-in-Human Phase I Study of an Oral HSP90 Inhibitor, TAS-116, in Patients with Advanced Solid Tumors. Mol. Cancer Ther..

[107] Felip E., Barlesi F., Besse B., Chu Q., Gandhi L., Kim S.-W., Carcereny E., Sequist L.V., Brunsvig P., Chouaid C. (2018). Phase 2 Study of the HSP-90 Inhibitor AUY922 in Previously Treated and Molecularly Defined Patients with Advanced Non-Small Cell Lung Cancer. J. Thorac. Oncol..

[108] Park H.-K., Yoon N.G., Lee J.-E., Hu S., Yoon S., Kim S.Y., Hong J.-H., Nam D., Chae Y.C., Park J.B. (2020). Unleashing the Full Potential of Hsp90 Inhibitors as Cancer Therapeutics through Simultaneous Inactivation of Hsp90, Grp94, and TRAP1. Exp. Mol. Med..

[109] Patel P.D., Yan P., Seidler P.M., Patel H.J., Sun W., Yang C., Que N.S., Taldone T., Finotti P., Stephani R.A. (2013). Paralog-Selective Hsp90 Inhibitors Define Tumor-Specific Regulation of HER2. Nat. Chem. Biol..

[110] Patel H.J., Patel P.D., Ochiana S.O., Yan P., Sun W., Patel M.R., Shah S.K., Tramentozzi E., Brooks J., Bolaender A. (2015). Structure-Activity Relationship in a Purine-Scaffold Compound Series with Selectivity for the Endoplasmic Reticulum Hsp90 Paralog Grp94. J. Med. Chem..

[111] Kirkland J.L., Tchkonia T., Zhu Y., Niedernhofer L.J., Robbins P.D. (2017). The Clinical Potential of Senolytic Drugs. J. Am. Geriatr. Soc..

